# Pentraxin 3 Levels Reflect Inflammatory and Parasitic Activity in Human Visceral Leishmaniasis

**DOI:** 10.3390/pathogens14121299

**Published:** 2025-12-18

**Authors:** Lucyo Flávio Bezerra Diniz, Milena Xavier Silva Barbosa, Samuel Ricarte de Aquino, Anderson da Costa Armstrong, Carlos Dornels Freire de Souza, Rodrigo Feliciano Carmo

**Affiliations:** 1 Postgraduate Program in Health and Biological Sciences, Federal University of the São Francisco Valley (UNIVASF), Petrolina 56304-917, Pernambuco, Brazil; lucyoflaviodiniz@gmail.com (L.F.B.D.); carlos.dornels@univasf.edu.br (C.D.F.d.S.); 2 University Hospital of the Federal University of the São Francisco Valley (HU-UNIVASF), Petrolina 56304-917, Pernambuco, Brazil; samricarte@hotmail.com (S.R.d.A.); anderson.armstrong@univasf.edu.br (A.d.C.A.); 3 Regional Hospital of Juazeiro, Juazeiro 48903-200, Bahia, Brazil; 4 Postgraduate Program in Applied Cellular and Molecular Biology, University of Pernambuco (UPE), Recife 50100-130, Pernambuco, Brazil; milenaxsb@gmail.com; 5 Advanced Laboratory for Diagnosis and Studies in Health and Environment (LADESA), College of Medicine, Federal University of the São Francisco Valley (UNIVASF), Petrolina, 56304-917, Pernambuco, Brazil; 6 Petrolina Municipal Health Secretariat, Petrolina 56304-020, Pernambuco, Brazil

**Keywords:** visceral leishmaniasis, Pentraxin 3, host–pathogen interaction, biomarker, inflammation, parasite load, therapeutic response

## Abstract

Visceral leishmaniasis (VL) is a severe zoonotic disease characterized by high mortality and a pronounced systemic inflammatory response. Although Pentraxin 3 (PTX3) has been implicated in infectious and inflammatory disorders, its role in human VL remains poorly defined, and host-derived indicators that simultaneously reflect inflammatory and parasitic activity are limited. This study investigated the association between plasma PTX3 levels, parasite load, and *PTX3* gene polymorphisms (rs1840680 and rs2305619) in patients with VL. An observational study was conducted between 2017 and 2021, including 36 patients with confirmed VL and 45 healthy controls matched by age and sex. Plasma PTX3 concentrations were determined by ELISA, parasite load by quantitative PCR (qPCR), and cytokines (IL-2, IL-6, IL-10, IL-17A, IFN-γ and TNF-α) by flow cytometry. PTX3 levels were significantly higher in VL patients than in controls (23.2 ng/mL vs. 0.80 ng/mL; *p* < 0.0001) and correlated positively with parasite load (r = 0.39; *p* = 0.02) and cytokines IL-6, IL-10 and IFN-γ. No associations were observed between *PTX3* polymorphisms and disease susceptibility. These findings suggest that PTX3 reflects both inflammatory responses and parasitic burden in VL and may serve as a potential indicator of disease activity.

## 1. Introduction

Visceral leishmaniasis (VL), also known as kala-azar, is a severe systemic zoonosis caused by protozoa of the *Leishmania* genus and transmitted through the bite of infected female sandflies (*Lutzomyia longipalpis*). In the Americas—and particularly in Brazil—VL is caused almost exclusively by *Leishmania infantum* (syn. *L. chagasi*), which is the only species associated with autochthonous transmission, including in the Northeast region. VL represents the most severe clinical form of leishmaniasis, with mortality exceeding 95% in untreated cases [1].

Globally, an estimated 50,000 to 90,000 new VL cases occur annually, resulting in approximately 5000 deaths, mainly in the Indian subcontinent, East Africa, and South America [1]. In Brazil, about 63,966 cases were reported between 2001 and 2020, with an average incidence of 1.6 cases per 100,000 inhabitants and a case fatality rate of 6.9% [2]. The Northeast region accounts for 45–55% of Brazilian cases, followed by the North and Southeast regions, with Maranhão, Piauí, Ceará, Bahia, and Pernambuco constituting major endemic areas [2,3,4]. In the Petrolina–Juazeiro axis, located in the semi-arid São Francisco Valley, VL displays a hyperendemic profile with increasing urbanization: during 2010–2016 incidence rates ranged from 2.0 to 8.6 cases per 100,000 inhabitants in Juazeiro and from 2.8 to 6.1 cases per 100,000 inhabitants in Petrolina. In that series, VL/HIV coinfection was 16.8% in Petrolina and 5.4% in Juazeiro, and cases predominated among young males and residents of rural or peri-urban areas [3].

Diagnosis relies on parasitological confirmation by microscopic examination of bone marrow or splenic aspirates, molecular detection of *Leishmania* DNA by qPCR performed on peripheral blood, and serological assays such as the rK39 immunochromatographic test (ICT) or ELISA. Quantification of parasite load by qPCR, beyond its diagnostic utility [5], has been explored as a marker of therapeutic response and relapse risk [6]. Treatment includes pentavalent antimonials, liposomal amphotericin B, amphotericin B deoxycholate or miltefosine, and its effectiveness may be monitored through parasitological and immunological biomarkers [7,8,9,10].

The pathophysiology of VL is characterized by intense systemic inflammation, with elevated mediators such as IL-6, IL-8, and IL-10 [10,11]. In this context, Pentraxin 3 (PTX3)—an acute-phase protein produced by myeloid and endothelial cells—has emerged as a potential biomarker of inflammatory activity, endothelial dysfunction, and disease prognosis [12,13,14,15]. PTX3 differs structurally and functionally from short pentraxins such as C-reactive protein (CRP), being synthesized locally at sites of infection by monocytes, macrophages, endothelial, and epithelial cells [12,13]. PTX3 participates in pathogen opsonization, complement activation, and modulation of inflammation [13].

*PTX3* polymorphisms (rs1840680 and rs2305619) have been associated with susceptibility to fungal, bacterial, and viral infections, including leprosy, aspergillosis, and COVID-19 [15,16,17]. These intronic variants may influence circulating protein levels and modulate ligand–receptor interactions [18]. In neglected tropical diseases, elevated plasma PTX3 has been described in multibacillary leprosy, with a marked decline after treatment [15]. In cutaneous leishmaniasis, PTX3 expression is increased in skin lesions and correlates with disease severity [19]. Given the immunopathological profile of VL−characterized by systemic inflammation, endothelial activation, and concomitant regulatory responses−PTX3 may serve as an indicator of host inflammatory status during infection.

Despite advances in understanding VL immunopathogenesis, no widely validated plasma marker currently integrates information on both inflammatory burden and parasite load. In this context, PTX3 has emerged as a molecule of potential interest, but its role in human VL remains insufficiently characterized. Additionally, little is known about the temporal kinetics of PTX3 after antigenic clearance in parasitic infections. Experimental and clinical data indicate that PTX3 rises rapidly within 6–8 h of inflammatory stimulation and typically declines over days following resolution of the triggering event, although persistence may occur in chronic or slowly resolving infections [12,20,21]. Such kinetics further support the rationale for investigating PTX3 in VL, a disease marked by sustained inflammation and high antigenic burden.

The aim of this study was to evaluate plasma PTX3 levels and *PTX3* gene polymorphisms (rs1840680 and rs2305619) in patients with VL and to examine their relationship with clinical, laboratory, and parasitological parameters compared with healthy controls.

## 2. Materials and Methods

### 2.1. Study Design

This observational analytical study included cross-sectional comparisons between visceral leishmaniasis (VL) patients and healthy controls, as well as paired pre- and post-treatment analyses in VL patients. Individuals were recruited between 2017 and 2021 from two reference hospitals in Northeastern Brazil. Both centers are reference for treatment of VL in the region, which receives approximately 10 cases per year. The control group consisted of healthy blood donors and community residents from the same endemic region, matched to cases by age and sex.

The study was conducted in accordance with the Declaration of Helsinki, and approved by the Ethics Committee of the Universidade Federal do Vale do São Francisco under the number CAAE:68562617.3.0000.5196. Informed consent was obtained from all subjects involved in the study.

### 2.2. Sample Size Calculation

Sample-size estimates for the primary case–control comparison (plasma PTX3 levels) were calculated a priori for a two-sided *t*-test (α = 0.05, power = 0.80). The standard deviation of PTX3 concentrations (1.24 ng/mL) was derived from the original raw data of a previously published cohort from the same population [15], by estimating the overall variability across all individuals (details provided in Supplementary Table S1). Assuming a mean difference of 1.0 ng/mL between groups (Cohen’s *d* ≈ 0.81), a minimum of 25 participants per group was required. To account for potential losses, the target sample size was increased to 30 cases and 30 controls.

### 2.3. Inclusion and Exclusion Criteria

Participants were eligible if they had a confirmed diagnosis of visceral leishmaniasis established by demonstration of *Leishmania* amastigotes by microscopy of bone marrow aspirate and/or a positive rK39 ICT obtained prior to initiation of specific anti-leishmanial therapy. According to Brazilian Ministry of Health recommendations [22], parasite visualization in bone-marrow aspirate is the diagnostic gold standard and was performed by experienced microscopists in the participating centers. The rK39 is accepted as supportive evidence in situations where bone-marrow aspirate is contraindicated or not feasible. For the present series, all enrolled patients had parasitological confirmation by bone marrow microscopy. Patients with HIV coinfection, autoimmune diseases, malignant neoplasms, or under immunosuppressive therapy were excluded from the study.

### 2.4. Sample Collection and Processing

Peripheral blood samples were obtained before and after anti-leishmanial therapy in accordance with Brazilian Ministry of Health guidelines [22]. Post-treatment samples were collected during the first clinical follow-up visit after completion of therapy, which varied according to therapeutic scheme. Therapeutic regimens included liposomal amphotericin B (total dose 21 mg/kg over 5–7 days), amphotericin B deoxycholate (1 mg/kg/day for 14–20 days), or pentavalent antimonials (20 mg Sb^5+^/kg/day for 20–30 days). Treatment allocation followed Brazilian Ministry of Health guidelines [22], which recommend different regimens based on clinical severity, age, comorbidities, renal/hepatic function, and drug availability in the hospital. Therefore, the observed diversity in therapeutic schemes reflects standard clinical practice rather than study-driven selection. Whole blood, serum and plasma aliquots were stored at −80 °C until analysis.

### 2.5. Parasite Load Quantification

Parasite load (parasites/mL) was quantified in peripheral blood by real-time PCR targeting kinetoplast DNA (kDNA), following the method described by Dantas-Torres et al. (2017) [5]. DNA extraction was performed using the PureLink™ Genomic DNA Mini Kit (Invitrogen, Carlsbad, CA, USA). Amplification was carried out on a QuantStudio™ 5 Real-Time PCR System (Applied Biosystems, Waltham, MA, USA) using primers LEISH-1 (5′-AACTTTTCTGGTCCTCCGGGTAG-3′) and LEISH-2 (5′-ACCCCCAGTTTCCCGCC-3′), together with a TaqMan-MGB probe (FAM-5′-AAAAATGGGTGCAGAAAT-3′-NFQ-MGB).

### 2.6. Determination of PTX3 Concentration

Plasma PTX3 concentrations were measured using a commercial ELISA kit (Human Pentraxin 3/TSG-14 Quantikine™, R&D Systems, Minneapolis, MN, USA), following the manufacturer’s instructions. Microplates were washed using an Asys Atlantis^®^ automated washer (Biochrom Ltd., Cambridge, UK), and absorbance was read at 450 nm (with 620 nm correction) using a Multiskan FC microplate reader (Thermo Fisher Scientific, Waltham, MA, USA). Concentrations were interpolated using a four-parameter logistic regression curve.

### 2.7. PTX3 Genotyping

Genotyping of *PTX3* polymorphisms rs1840680 (A > G) and rs2305619 (A > G) was performed using genomic DNA extracted with the PureLink™ Genomic DNA Mini Kit (Invitrogen, Carlsbad, CA, USA). DNA purity and concentration were assessed using a NanoDrop OneC spectrophotometer (Thermo Fisher Scientific, Waltham, MA, USA), and samples with A260/A280 ratios between 1.7 and 2.0 were included.

Real-time PCR reactions were performed on a QuantStudio™ 5 System using TaqMan^®^ SNP Genotyping Assays (Applied Biosystems, Waltham, MA, USA). Assay IDs were C__25475188_10 for rs1840680 and C__15879983_10 for rs2305619, with VIC^®^ (allele A) and FAM^®^ (allele G) probes. The thermal protocol consisted of 50 °C for 2 min, 95 °C for 10 min, followed by 40 cycles of 95 °C for 15 s and 60 °C for 1 min. Allelic discrimination was conducted using TaqMan Genotyper™ Software v1.6. Genotypic distributions were tested for Hardy–Weinberg equilibrium (*p* > 0.05), and allele/genotype frequencies were compared between groups.

### 2.8. Cytokine Quantification

Serum concentrations of IL-2, IL-6, IL-10, IL-17A, IFN-γ, and TNF-α were measured in pre-treatment samples using cytometric bead array (CBA) kits (BD Biosciences, San Diego, CA, USA), following the manufacturer’s protocol. Acquisition was performed on a BD™ FACSCalibur flow cytometer, and data were analyzed using FCAP Array™ Software version 1.0.1.

### 2.9. Statistical Analysis

Statistical analyses were performed using JASP (version 0.19.0) and GraphPad Prism 8.0 (GraphPad Software, San Diego, CA, USA). Normality was assessed using the Shapiro–Wilk test. Normally distributed variables were expressed as mean ± standard deviation (SD) and compared using Student’s *t*-test. Non-parametric variables were expressed as median and interquartile range (IQR) and compared using the Mann–Whitney U or Kruskal–Wallis tests, as appropriate. Correlations were evaluated using Spearman’s rank correlation coefficient. Genotype frequencies were compared using the chi-square test. Statistical significance was set at *p* < 0.05.

## 3. Results

### 3.1. Clinical and Laboratory Characteristics

A total of 36 patients with visceral leishmaniasis (VL) and 45 age- and sex-matched healthy controls were included. The mean age of VL patients was 39.4 ± 17.0 years, while the controls had a mean age of 33.7 ± 10.7 years. Males predominated in both groups (88.8%) (Table 1).

Among VL patients, the most frequent clinical manifestations were asthenia (88.8%) and fever (83.3%), followed by abdominal pain (52.7%) and bleeding episodes (27.7%). Comorbidities occurred at lower frequencies, including kidney disease (11.1%), liver disease (8.3%), and heart disease (2.7%). The case-fatality rate was 11.1% (Table 1).

Regarding treatment, liposomal amphotericin B (ABL) was the most commonly used regimen (38.8%), followed by meglumine antimoniate (Glucantime^®^, 25.0%) and amphotericin B deoxycholate (8.3%). Sequential therapeutic schemes included Glucantime^®^ followed by amphotericin B (13.8%), amphotericin B deoxycholate followed by Glucantime^®^ (8.3%), and amphotericin B deoxycholate followed by liposomal amphotericin B (5.5%) (Table 1).

### 3.2. Plasma PTX3 Levels in VL Patients and Controls

**Figure 1:**
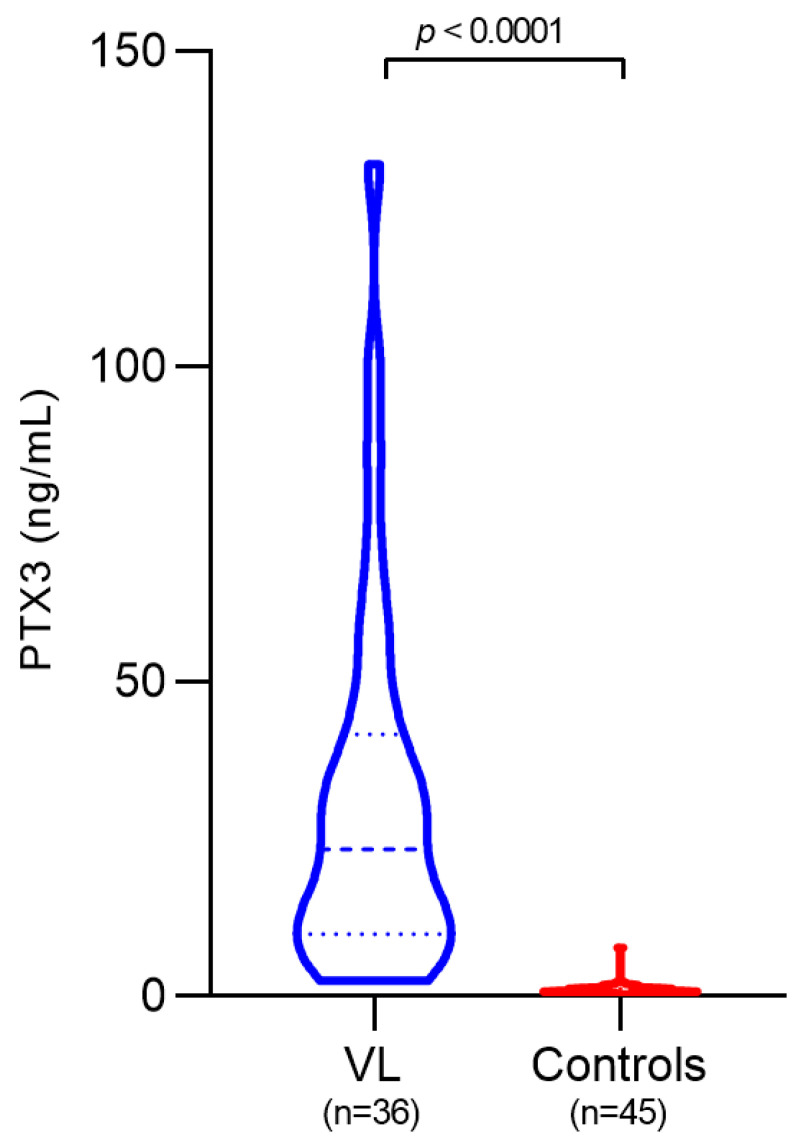
Plasma pentraxin-3 (PTX3) levels in VL patients and healthy controls. Mann–Whitney U test.

Pre-treatment plasma PTX3 levels were markedly elevated in VL patients compared with healthy controls (median: 23.2 ng/mL vs. 0.80 ng/mL; *p* < 0.0001), representing an approximately 29-fold increase (Figure 1).

### 3.3. Plasma PTX3 Levels Before and After Treatment

**Figure 2:**
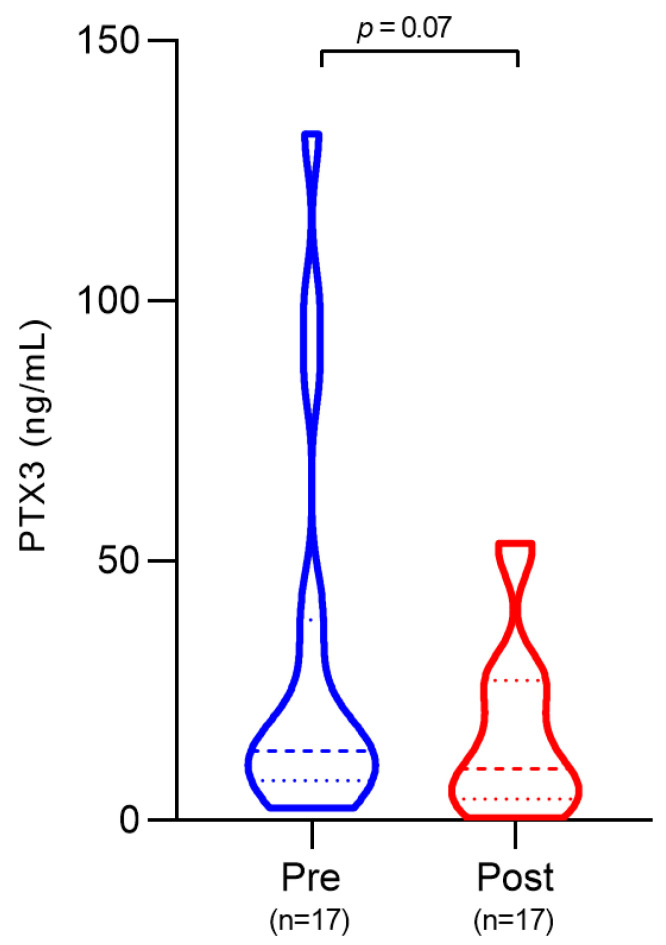
Plasma PTX3 levels in VL patients before and after anti-leishmanial treatment. Wilcoxon matched-pairs test.

Among the subset of patients with paired samples (*n* = 17), PTX3 was elevated during active disease (median: 13.36 ng/mL) and decreased after treatment (median: 10.02 ng/mL). Although a downward trend was observed, the reduction did not reach statistical significance (*p* = 0.07) (Figure 2). Post-treatment peripheral blood qPCR results were available for the majority of patients (*n* = 15). Among these, two patients had undetectable Leishmania kDNA at the immediate post-treatment time point, whereas 13 patients remained qPCR-detectable. Patients with undetectable parasitemia showed markedly lower PTX3 concentrations after therapy compared with patients who remained qPCR-positive (median PTX3: 1.545 ng/mL vs. 17.70 ng/mL; *p* = 0.01) (Supplementary Figure S1). These subgroup data suggest that PTX3 levels may fall more substantially in patients who achieve early parasitological clearance, but the small number of patients with undetectable parasite limits a definitive conclusions.

### 3.4. Correlation Between PTX3 and Parasite Load

**Figure 3:**
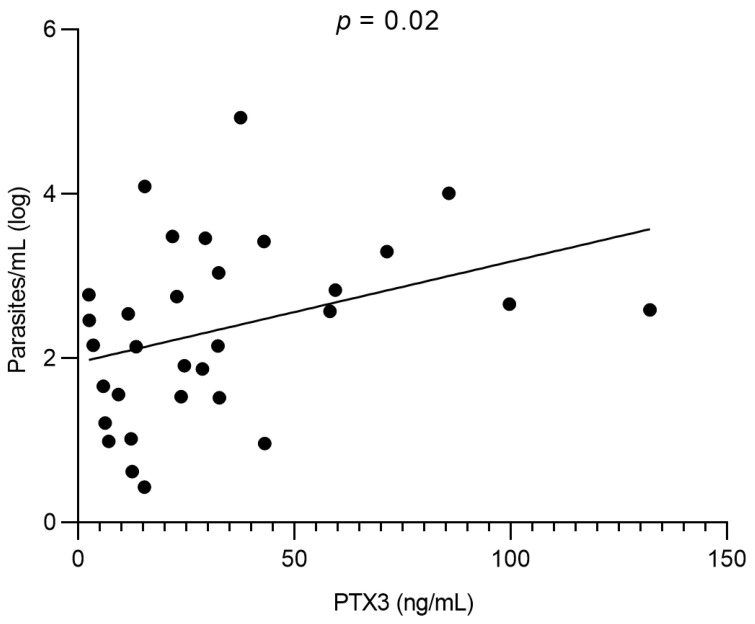
Correlation between plasma PTX3 and parasite load in VL patients. Spearman’s correlation (r = 0.39).

The mean parasite load quantified by qPCR was 2.1 log parasites/mL (range: 0.4–4.9). Pre-treatment PTX3 levels showed a significant positive correlation with parasite load (r = 0.39; *p* = 0.02), indicating that higher PTX3 concentrations accompany greater parasitemia (Figure 3).

### 3.5. PTX3 Gene Polymorphisms

Genotypic distributions for rs1840680 and rs2305619 were in Hardy–Weinberg equilibrium in both groups. For rs1840680, genotype frequencies in VL patients were GG (33.3%), AG (52.8%), and AA (13.9%), compared with GG (42.2%), AG (35.6%), and AA (22.2%) in controls.

For rs2305619, frequencies in VL patients were GG (19.4%), AG (52.8%), and AA (27.8%), compared with GG (33.3%), AG (40.0%), and AA (26.7%) in controls.

No significant differences were observed between cases and controls for either polymorphism (*p* > 0.05; Table 2).

### 3.6. Correlation Between PTX3 and Inflammatory Cytokines

**Figure 4:**
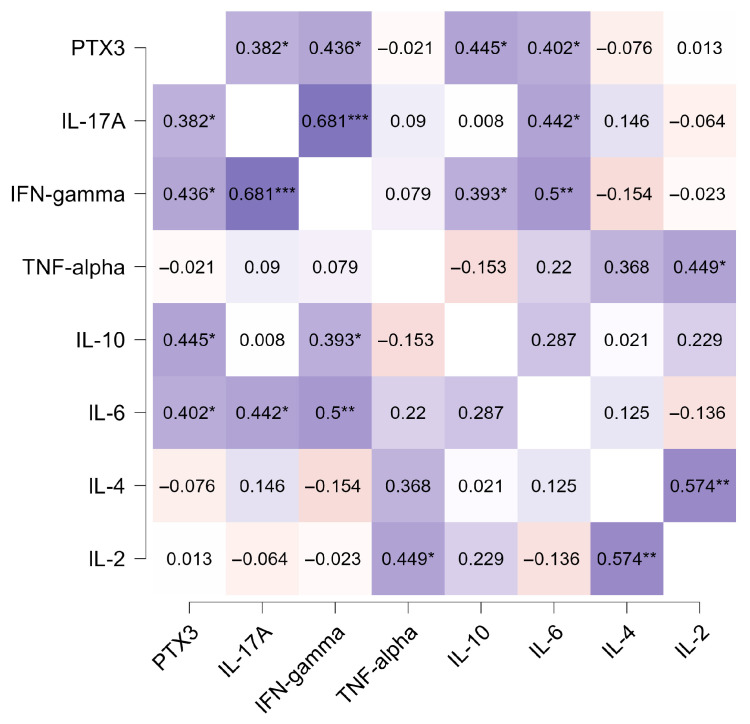
Correlation between plasma PTX3 levels and inflammatory cytokines in VL patients. Numbers represent Spearman rho values. * *p* < 0.05, ** *p* < 0.01, *** *p* < 0.001.

Correlation analyses revealed heterogeneous associations between PTX3 and inflammatory mediators. PTX3 levels correlated positively with IL-6 and IL-10, suggesting an association with systemic inflammatory activation. Significant positive correlations with IL-17A and IFN-γ were also observed, indicating that PTX3 reflects both pro-inflammatory and regulatory immune pathways. No significant correlations were detected for IL-2 or TNF-α (Figure 4).

## 4. Discussion

The present study demonstrates that PTX3 is markedly elevated during active VL and shows a tendency to decrease following treatment, supporting its role as a dynamic marker of disease activity. Although longitudinal data in human VL are scarce, our findings align with evidence from other infectious diseases showing that PTX3 reflects systemic inflammation and, in some settings, treatment response. In multibacillary leprosy, Moraes et al. (2023) [15] reported a significant reduction in PTX3 levels after multidrug therapy, mirroring declines in bacterial load. The same pattern was observed in *Mycobacterium tuberculosis* infections, where PTX3 plasma levels were significantly decreased in individuals who responded to therapy compared to patients with treatment failure [23], and in patients with community-acquired pneumonia after antibiotic treatment [24]. These studies support the broader understanding that PTX3 peaks early in response to inflammatory stimuli and gradually declines as antigenic burden and tissue injury resolve, consistent with known PTX3 kinetic behavior described in the literature [12,20,21].

Importantly, our study benefits from the availability of paired parasite quantification by peripheral blood qPCR in most patients and is concordant with prior longitudinal work from the same setting showing rapid reduction in blood parasite load after therapy. Aquino et al. (2023) reported a significant decrease in blood *Leishmania* kDNA immediately after treatment (one day after treatment completion), although the majority of patients remained qPCR-positive at that early time point and many only cleared parasitemia at subsequent visits (3 months or later) [6]. Because most post-treatment samples in our current study were collected early after therapy completion, this timing likely explains why the paired PTX3 reduction failed to reach statistical significance (*p* = 0.07) despite a downward trend. PTX3 is expected to decrease as antigenic stimulus and tissue injury resolve, but the decline may lag behind clinical improvement and depends on the speed of parasite clearance.

Consistent with this interpretation, the subset analysis presented here shows that patients who already had undetectable blood parasitemia immediately after treatment exhibited substantially lower PTX3 concentrations than patients who remained qPCR-positive, suggesting that PTX3 levels are sensitive to residual parasitic burden. These observations are consistent with the parasite clearance kinetics reported by Aquino et al. (2023) [6] and suggest that PTX3 may be more closely aligned with parasitological resolution than with clinical recovery alone.

Taken together, these observations suggest that the timing of post-treatment sampling is critical when assessing PTX3 dynamics. Early post-treatment evaluation may capture partial rather than full resolution of PTX3, whereas later time points (e.g., >3 months) may better reflect complete normalization following parasitological clearance. Longitudinal sampling at multiple standardized time points is therefore required to fully characterize PTX3 kinetics in VL and to establish optimal windows for its use as a monitoring biomarker.

Experimental evidence also supports a functional role for PTX3 in leishmanial infections. In cutaneous leishmaniasis, Gupta et al. (2020) [19] showed that PTX3 is highly expressed in lesions and that PTX3-deficient mice infected with *L. major* develop smaller lesions and lower parasite burdens. This enhanced resistance was associated with stronger IL-17A/Th17 responses, whereas IFN-γ–mediated Th1 responses were not substantially affected. Although derived from cutaneous disease, these findings support the biological plausibility that PTX3 may modulate host inflammatory responses in visceral leishmaniasis as well.

In our cohort PTX3 correlated positively with peripheral *Leishmania* load, suggesting that PTX3 levels reflect active infection. Although studies that directly link PTX3 to quantified parasitemia in humans are limited, elevated PTX3 has been associated with greater clinical severity or worse outcomes in a range of infectious diseases (e.g., pulmonary aspergillosis, tuberculosis, dengue, meningococcal disease, leptospirosis, shigellosis) [20,23,25,26,27,28,29], supporting the view that PTX3 tracks inflammation intensity in severe infections. Cross-sectional malaria studies [30,31] report higher PTX3 concentrations in severe or cerebral disease compared with milder forms, consistent with PTX3 as a marker of inflammatory activation in parasitic infections.

Correlations between PTX3 and cytokines such as IL-6, IL-17A, IFN-γ, and IL-10 underscore the pleiotropic nature of this molecule and its involvement in distinct immunological pathways. These cytokines represent complementary arms of the host response in VL, spanning pro-inflammatory (IL-6), Th1-mediated effector responses (IFN-γ), Th17-associated activity (IL-17A), and immunoregulatory mechanisms (IL-10). Evidence from human VL studies shows that dysregulated IFN-γ responses and elevated IL-10 contribute to disease progression, whereas restoration of IFN-γ production accompanies clinical recovery [32,33]. IL-17A has also been implicated in VL pathogenesis, with studies demonstrating that IL-17A participates in inflammatory activation and may act synergistically with IFN-γ to enhance parasite control [34]. These observations are consistent with Araújo-Santos et al. [10], who showed that anti-parasite therapy partially restores inflammatory balance by reducing IL-6 and IL-10 during treatment. Together, the cytokine correlations we observed suggest that PTX3 functions as an integrative marker of both pro- and anti-inflammatory circuits in VL, a dual role previously described for PTX3 in other infectious and inflammatory contexts [35].

From a mechanistic standpoint, PTX3 contributes to humoral innate immunity through roles in pathogen recognition, complement activation, opsonization, and tissue remodeling [21]. Its interactions with C1q, factor H, and other complement components place PTX3 at a central position in regulating inflammatory cascades [36], explaining its consistent upregulation across infectious diseases.

We did not observe an association between *PTX3* polymorphisms rs1840680 and rs2305619 and VL. While *PTX3* variants have been linked to susceptibility or outcome in some infections (tuberculosis [37], aspergillosis [16], pneumonia [38], COVID-19 [17]), such effects appear to be context- and pathogen-dependent. Our negative findings may be explained by the limited sample size, which restricts statistical power to detect modest genetic effects. Larger cohorts are therefore required to confirm this result.

Importantly, to our knowledge, this is the first study to assess plasma PTX3 concentrations together with *PTX3* gene polymorphisms in human visceral leishmaniasis. By integrating clinical, inflammatory, parasitological, and genetic data, our findings provide the first direct evidence that circulating PTX3 levels reflect both inflammatory activation and parasite burden in VL. These observations support the possibility that PTX3 may serve as a complementary laboratory indicator, potentially contributing to the assessment of disease activity and therapeutic response when used alongside established tools such as qPCR.

This study has limitations, including a modest sample size, incomplete longitudinal follow-up, and limited statistical power for detecting genetic associations. Nevertheless, the consistency of our findings and their biological plausibility suggest that PTX3 warrants further evaluation in the context of VL. Larger, multicenter, and fully longitudinal studies are needed to determine the reproducibility, prognostic utility, and clinical applicability of PTX3 in patient monitoring and risk stratification.

## 5. Conclusions

PTX3 concentrations were markedly elevated during active visceral leishmaniasis and showed a trend toward decline after treatment. Importantly, patients who achieved undetectable blood parasitemia at the immediate post-treatment time point exhibited significantly lower PTX3 concentrations than those who remained qPCR-positive, suggesting that PTX3 levels reflect residual parasitic burden as well as inflammatory activity. This study provides the first human data linking circulating PTX3 with both inflammatory mediators and peripheral parasite load in VL and supports the potential role of PTX3 as a complementary laboratory indicator. However, these findings must be considered preliminary given the small sample size and the early timing of many post-treatment samples; larger, multicenter, and longitudinal studies with standardized sampling at multiple time points are required to validate PTX3’s prognostic value and to determine how best to integrate PTX3 with qPCR and cytokine panels for clinical monitoring and decision-making in endemic settings.

## Figures and Tables

**Table 1:** Demographic and clinical characteristics of patients with visceral leishmaniasis (VL) and healthy controls.

Variable	VL (*n* = 36)	Controls (*n* = 45)
Demographic characteristics		
Age (years) *, mean ± sd	39.4 ± 17.0	33.7 ± 10.7
Male sex *, *n* (%)	32 (88.8)	40 (88.8)
Clinical characteristics		
Asthenia, *n* (%)	32 (88.8)	0
Fever, *n* (%)	30 (83.3)	0
Abdominal pain, *n* (%)	19 (52.7)	0
Bleeding, *n* (%)	10 (27.7)	0
Kidney disease, *n* (%)	4 (11.1)	0
Liver disease, *n* (%)	3 (8.3)	0
Heart disease, *n* (%)	1 (2.7)	0
Death, *n* (%)	4 (11.1)	0
Treatment scheme		
ABL, *n* (%)	14 (38.8)	0
GLU, *n* (%)	9 (25.0)	0
ADC, *n* (%)	3 (8.3)	0
GLU followed by ABL, *n* (%)	5 (13.8)	0
ADC followed by GLU, *n* (%)	3 (8.3)	0
ADC followed by ABL, *n* (%)	2 (5.5)	0

Legend: ABL, Amphotericin B Liposomal; GLU, Glucantime; ADC, Amphotericin B Deoxycholate. * *p* > 0.05.

**Table 2:** Distribution of *PTX3* gene polymorphisms (rs1840680 and rs2305619) in patients with visceral leishmaniasis (VL) and healthy controls.

	VL (*n* = 36)	Controls (*n* = 45)	*p*-Value *
*rs1840680*			
GG, *n* (%)	12 (33.3)	19 (42.2)	0.28
AG, *n* (%)	19 (52.8)	16 (35.6)	
AA, *n* (%)	5 (13.9)	10 (22.2)	
*rs2305619*			
GG, *n* (%)	7 (19.4)	15 (33.3)	0.34
AG, *n* (%)	19 (52.8)	18 (40.0)	
AA, *n* (%)	10 (27.8)	12 (26.7)	

Legend: *p*-values were calculated using the chi-square test comparing genotype distributions between VL patients and controls. All genotype distributions were in Hardy–Weinberg equilibrium (* *p* > 0.05).

## Data Availability

The data presented in this study are available on request from the corresponding author due to ethical restrictions.

## Supplementary Materials

The following supporting information can be downloaded at: https://www.mdpi.com/article/10.3390/pathogens14121299/s1, Figure S1: Plasma PTX3 concentrations in visceral leishmaniasis patients stratified according to post-treatment peripheral blood parasite DNA detectability by qPCR. Mann–Whitney U test; Table S1: Individual-level plasma PTX3 concentrations (ng/mL) in leprosy patients, household contacts, and blood donors used to calculate the pooled standard deviation for sample size estimation. These raw data correspond to the dataset underlying Moraes et al. (2023) [15] and were used to derive summary statistics (mean and pooled SD) applied in the a priori sample size calculation of the present study.
