# Supplementary figures and images for: Pentraxin 3 Levels Reflect Inflammatory and Parasitic Activity in Human Visceral Leishmaniasis
Source: Pathogens. 2025 Dec 18;14(12):1299. doi: 10.3390/pathogens14121299 (PMC12736011; doi:10.3390/pathogens14121299)

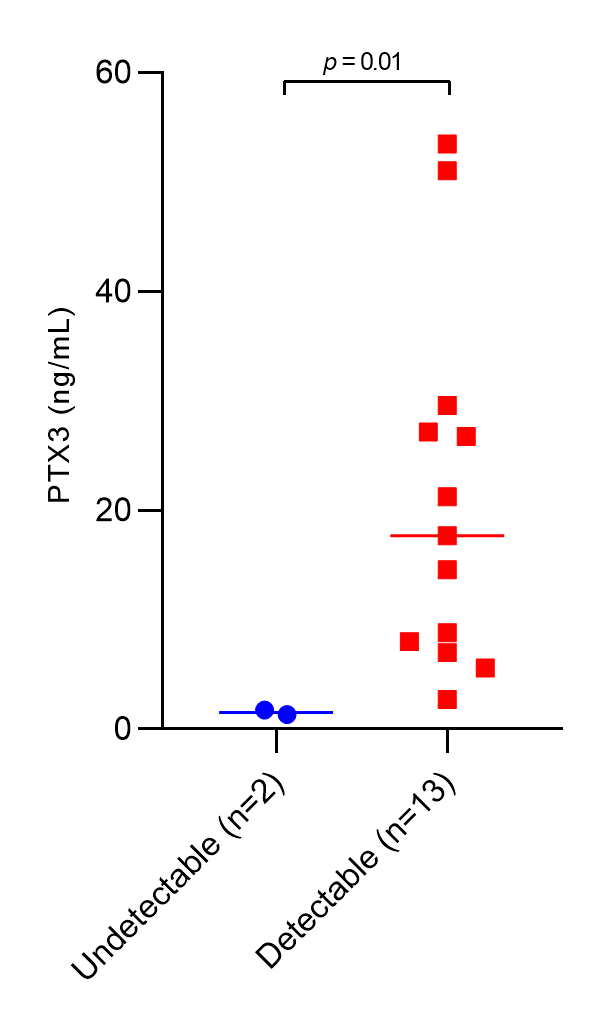

Supplement: Supplementary file 1 [file pathogens-14-01299-s001.zip › pathogens-4023875-supplementary/Supplementary figure 1.tif]
